# Comparative study on scenarios for rotating gantry mechanical structures

**DOI:** 10.12688/openreseurope.14683.1

**Published:** 2022-05-06

**Authors:** Luca Piacentini, Luca Dassa, Diego Perini, Andris Ratkus, Toms Torims, Stefano Uberti, Janis Vilcans, Maurizio Vretenar

**Affiliations:** 1 Center of High-Energy Physics and Accelerator Technologies,, Riga Technical University, Riga, LV-1048, Latvia; 2 Conseil Européen pour la Recherche Nucléaire, Geneva 23, 1211, Switzerland; 3 Department of Mechanical and Industrial Engineering, University of Brescia, Brescia, 25123, Italy

**Keywords:** Particle Therapy, Ion Therapy, Gantry, Mechanical Design

## Abstract

**Background:** This paper outlines a comparative study on scenarios of gantries mechanical design for ion-therapy of cancer, which is a crucial step toward the development of the next ion medical machine.

**Methods:** Multiple scenarios were considered based on robustness of the design, size, weight and complexity, deformation and precision performances and costs, as well as environmental impact. Four prospective scenarios were identified, each of them capable of providing beam to at least 220°around the patient. One scenario is capable of providing treatment angles of 360°. This report will describe the unified methodology performed during the study in order to achieve unbiased results in a comprehensive manner.

**Results:** Results show that in statically balanced scenarios, considerable improvements can be reached in terms of safety, deformation, precision performances, complexity and costs of implementation.

**Conclusions:** All scenarios are deemed suitable for further gantry design development.

## Introduction

In 2020, 2.7 million people in the European Union were diagnosed with cancer, and another 1.3 million people died as consequence of the same disease. Today, Europe hosts a tenth of world’s population but contributes to a quarter of world’s cancer cases. Figures are expected to rise by more than 24% by 2035. Although cancer is an individual diagnosis, it has non-negligible impacts on the lives of the patient’s relatives and social group. The overall economic impact of cancer in Europe is estimated to cost more than €100 billion per year ^1^.

In addition to traditional cancer treatments such as surgery, chemotherapy, immunotherapy and classical radiation therapy, during the last 60–70 years hadron therapy emerged as a new field in radiation therapy. In particular it demonstrated to be a key option for treatments of pediatric solid tumours, tumours of the brain, skull base, spinal cord, upper respiratory tract, chest, pelvic and others ^2^.

CERN is active in knowledge and technology transfer from physics to medicine and society in general. In 1996 the Proton-Ion Medical Machine Study (PIMMS) ^3, 4^ group was formed with commitment to design a cancer therapy synchrotron. CERN agreed to host this study in its Proton Synchrotron Division. One of the many results of this study has been the implementation of a synchrotron and a fixed gantry in the medical environment of the Italian National Center of Oncological hadron therapy (CNAO) and MedAustron in Austria.

With the definition of the European Strategy for Particle Physics in 2006, in one of its statements, CERN Council recognized the need to promote the impact of particle physics research on society proposing ‘to create a technology transfer forum to analyse the keys to the success in technology transfer projects in general’ ^5^. Since 2006 regular updates have been required by the European Strategy Group. Environmental and societal impact has been one of the pillars in every update, the most recent being in 2020 ^6^. Meanwhile, to further formalize the commitment of CERN in knowledge and technology transfer in medical applications, in 2014 the CERN Medical Applications Steering Group was founded by council decision, being this the very first organizational structure created with this scope. In 2016 a more robust structure was established ^7^ guided by CERN Medical Applications Steering Commitee.

As CERN did with PIMMS in the past, its commitment to develop next-generation ion therapy facilities resulted in the establishment of the Next Ion Medical Machine Study (NIMMS) ^8^, an initiative funded by the CERN Knowledge Transfer for Mediacl Applications and intended to stimulate wide European collaborations. NIMMS is organised in three work packages (superconducting magnets, linacs, gantries). The aim of the gantries work package is to design a superconducting ion gantry that is more efficient in term of costs and size with respect to existing ones, both normal-conducting and superconducting. Indeed, ion therapy requires a rotating gantry to maximize its efficacy giving to clinicians a tool that is able to provide 3D optimized treatments, necessary for example to better spare an organ at risk or to minimize the radiation dose in healthy surrounding tissues. Existing gantries for heavy ions, are massive (300 tons for the superconducting unit of HIMAC in Chiba ^9^ and 600 tons for the normal-conducting one of HIT in Heidelberg ^10^) and require high standard technologies, thus expensive. The cost makes these facilities a luxurious investment for countries, limiting general public accessibility to such treatments.

In the development of gantry technologies two projects approved and funded by European Commission Horizon 2020 Research Infrastructure are integrating the NIMMS resources with those of collaborating institutes throughout Europe, namely: HITRI *plus* (Heavy Ion Therapy Research Integration) ^11^ for the development of ion therapy accelerator technologies, and I.FAST (Innovation Fostering in Accelerator Science and Technology) for the development of curved superconducting magnets. These activities are additionally supported by the State Research Programme in High Energy Physics and Accelerator Technologies of Latvia ^12^. The combined efforts of these projects aim to answer the needs of the medical accelerator community for latest state of the art technologies associated to ion therapy.

Main developments on rotating isocentric gantries before the start of HITRI *plus* in April 2021 were concentrated on an innovative scenario named SIGRUM (Superconducting Ion Gantry with Riboni’s Unconventional Mechanics ^13, 14^), developed by TERA Foundation in collaboration with CERN. A following study was mainly focused on a possible mechanical optimization of the structure ^15^.

This note reports on a new collaborative effort between (CERN EN-MME, TE-MSC), RTU and UNIBS, which is carried out in consultancy with experts from the CNAO ion therapy centre as part of the HITRI *plus* project. Several scenarios of mechanical structures for isocentric gantries have been identified, a series of parameters to evaluate the compliance to main requirements for the selected scenarios have been defined and eventually the result of the comparison is reported.

### Challenges

The first major challenge is the control of the cost of the complete and functional machine that has to remain within some price limits defined in the HITRI *plus* project ^16^. Since a reduction in the load carried by the mechanical structure is expected from the use of bent superconducting magnets of new design, there are opportunities to reduce weight and cost. Moreover, a second challenge is to provide a comprehensive and impartial analysis to obtain reliable results while still in a preliminary phase of the project. The multidisciplinary nature of the gantry is in itself another relevant challenge, requiring to acknowledge interdisciplinary connections and to evaluate their influence on studied parameters. In general terms, the improvement of the Technology Readiness Level (TRL) ^17^ of this design is the final outcome of the project, from the presently evaluated TRL 2 for the mechanical structure and its motion system, whose technology concepts have been established.

### Main requirements

Table 1 summarizes the main requirements for the next generation of rotating gantries.

**Table 1.** List of relevant requirements for a rotating gantry.

**Safety**	robustness of the design
**Technological prerequisites**	size weight and complexity performances: deformation and precision
**Environmental**	minimal energy consumption
**Clinical requirements**	treatment angle coverage

Design robustness is guaranteed by relevant mechanical design choices. This requirement obviously comes as first rule while designing a machine or component that meets safety specifications.

Size requirements were set in accordance with CNAO based on expected available on-site space for a possible first implementation of the gantry, corresponding to a 20×20×20m ^3^ room (width × length × height). The HITRI *plus* project has placed specific requirements on weight and complexity to favour a subsequent industrialization of the design. The target in this case is to maintain the weight at about 100 tons, which is a substantial reduction when compared to the 600 and 300 tons for HIT and HIMAC respectively. Deformation and precision performances requirements are obtained by translating the needs of the medical community to ensure the proper accuracy in the beam delivery to the patient.

Environmental requirements, mainly related to the minimization of energy consumption, shall be considered as well. The machine consumes energy in multiple systems: cryogenic, transfer line magnets, accelerator magnets and gantry motion. However, the consumption of the latter is orders of magnitude lower than the others; its relevance is proportionally weighted in our study, since it is not heavily impacting the total efficiency of the system.

Important clinical requirements come from the necessity of maximizing effectiveness of treatment plans by using multiple treatment angles, thus a gantry rotation between ±110° and ±180° (measured from the horizontal plane) is required.

## Methods

The study has been done using a comprehensive approach aiming at being impartial in providing objective tools to compare mechanics advantages and disadvantages of each proposed scenario. This note reports about challenges related to the mechanical structure and its motion system, without considering aspects related to medical treatment. However, general inputs from clinicians have been considered, such as: estimated size of patient couch and its motion, desired volumetric imaging system at the isocenter and the gantry angular speed. The study was subdivided into three steps:

- Definition of relevant scenarios,
- Definition of relevant comparison parameters,
- Analysis of selected parameters.

This study has evolved directly from the SIGRUM analysis ^13, 14^. The proposal of scenarios was carried out during periodic meetings. Comparison parameters were proposed by taking into account the ones related to project requirements and challenges, with the expansion or addition of new parameters. The analysis of such parameters has been done by using the following tools:

- 3D modelling and Finite Element Analysis (FEA) software, Inventor and Solidworks,
- Computation software, Wolfram Mathematica and Microsoft Excel.

Results or hypotheses have been validated by double checking with different methods in the case of quantitative data (i.e. for mathematical models) or, if qualitative, by reaching common agreement during constant discussions within the group of experts mentioned above. Results have been summarized in a final Table by ranking the scenarios with respect to each parameter. The arbitrary ranking system consists in levels from one to five, related to a rising level of compliance to the specific parameter. During the study development, the awareness of crucial medical needs for appropriate treatment was always present.

## Comparison and analysis of scenarios for rotating gantry

The transfer line proposed in 13, 14 was used as general input for this study:

- 3T magnets,
- Overall radial position of the center of gravity of the line: 3.5m,
- Mass of the line: ≃ 12 ton.

The comparison of scenarios was done assuming independence of the mechanical structures from the transfer line general layout unless its mass and radial position of the center of gravity varies much (i.e. using 5T magnets). However, even in that case it is believed that relative advantages and disadvantages would not change much between scenarios. Between 10 and 15 scenarios, considering both balanced and unbalanced variants were proposed during discussion sessions with experts. However, not every scenario satisfied all general technological requirements (i.e. design robustness, energy consumption or size limits). Therefore, a screening process has been applied, mainly driven by considerations related to the robustness of the design and failure scenarios:

1. Scenarios can be categorized as ‘balanced’ (statically), if their center of gravity stands on the rotation axis, or ‘unbalanced’ otherwise. Balanced structures are achieved by the integration of a counterweight. All scenarios proposed in Table 2 are fully balanced.
2. The same equation of motion has been applied to simulate the movement of the gantry (three step constant acceleration-constant speed-constant deceleration). The time to make half a rotation has been assumed to be 30s, while any other rotation time is proportional. Thus, the maximum angular velocity is approximately 0.21 rad s ^−1^, independently for balanced and unbalanced.
3. Unbalanced scenarios can behave like physical pendulums in high danger situations (i.e. caused by lost of braking action, lost of hydraulic pressure, mechanical failure of ropes). Hence, the system accelerates due to gravity, the worst case being when the system is already at its maximum speed and a high danger failure occurs. It has been assumed that the reaction time of the braking system is 0.2 s and that balanced structures have double the moment of inertia with respect to unbalanced. Since the speed of unbalanced structures doubles during the reaction time, the rotational kinetic energy increase overall by a factor of two, with respect to balanced scenarios.

**Table 2.** Prospective scenarios of mechanical structure for rotating isocentric gantries.

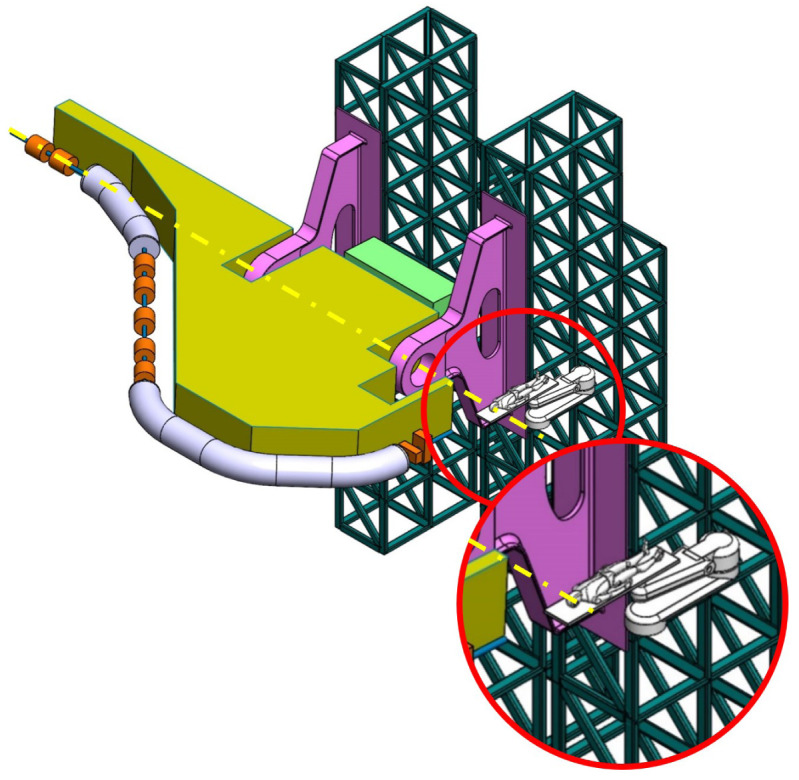	**Balanced SIGRUM (B-SIGRUM)** A balanced structure with two side supports, driven by an electric motor (and gearbox) directly mounted on the axis. Proposed to improve robustness of the design of SIGRUM only by the addition of a relatively simple counterweight (in green). • ±110° deliverable treatment angle range. • External volume of construction: 21.5 × 28.5 × 27m ^3^ • Internal volume of construction: 11.5 × 20.5 × 20m ^3^ • Rotating mass about 70 ton. • Relevant moment of inertia: 500000 kgm ^2^
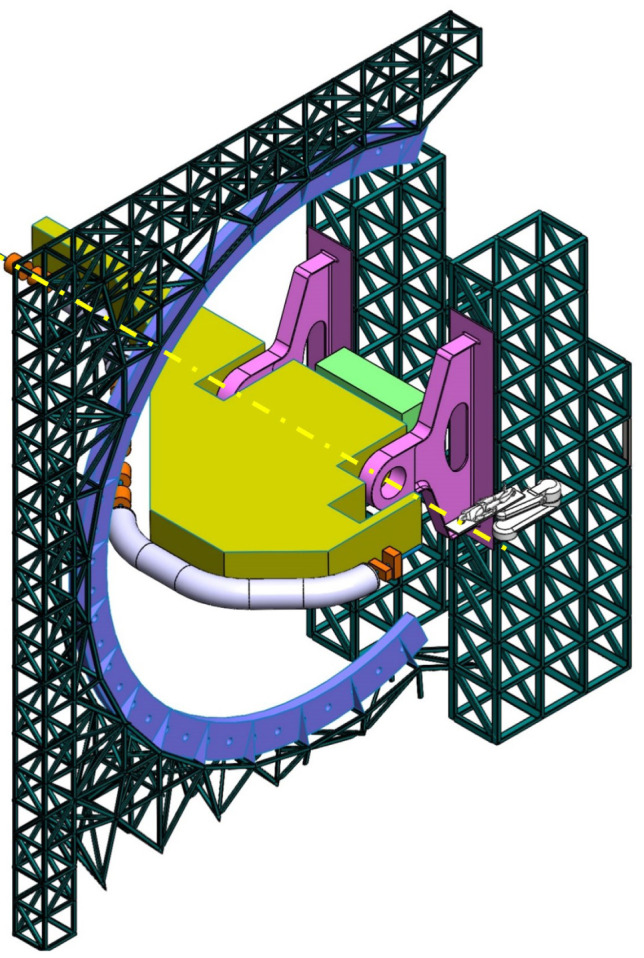	**Simply Supported Gantry (SSG)** Two side supports and one perimetrical support balanced structure. Driven by an external electric motor through off-axis rope and pulley transmission system. Proposed to reduce absolute displacement without unconventional compensation methods. • ±110° deliverable treatment angle range. • External volume of construction: 22×28.5× 30m ^3^ • Internal volume of construction: 12×20.5× 23m ^3^ • Rotating mass about 70 ton. • Relevant moment of inertia: 500000 kgm ^2^
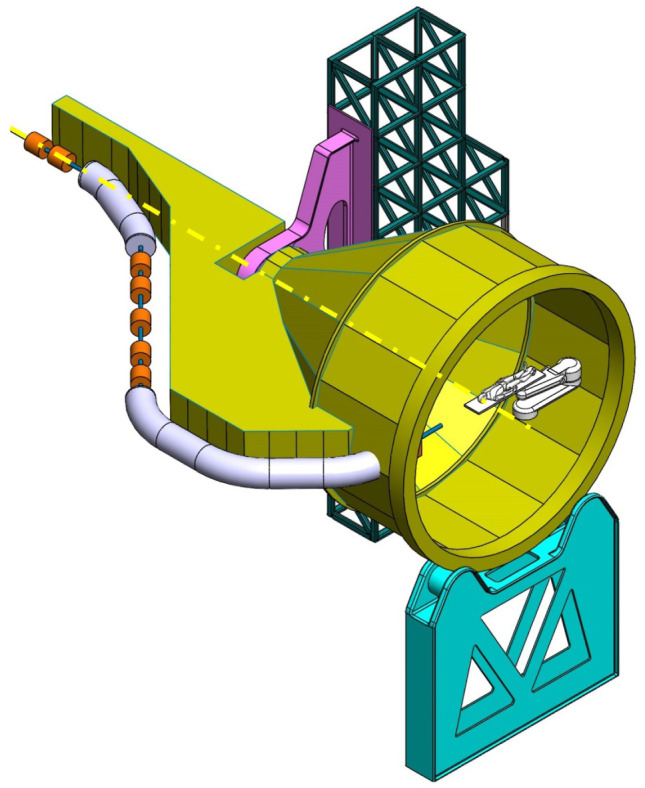	**Side and Cradle Supported Gantry (SCSG)** One side and one ground support balanced structure. Driven by off-axis friction rollers, coupled to an electric motor, or by external hydraulic system through rope transmission system. Proposed as intermediate solution between SIGRUM and Full Turn Gantry. • ±110° deliverable treatment angle range. • External volume of construction: 21.5 × 28.5 × 27m ^3^ • Internal volume of construction: 11.5 × 20.5 × 20m ^3^ • Rotating mass about 120 ton. • Relevant moment of inertia: 700000 kgm ^2^
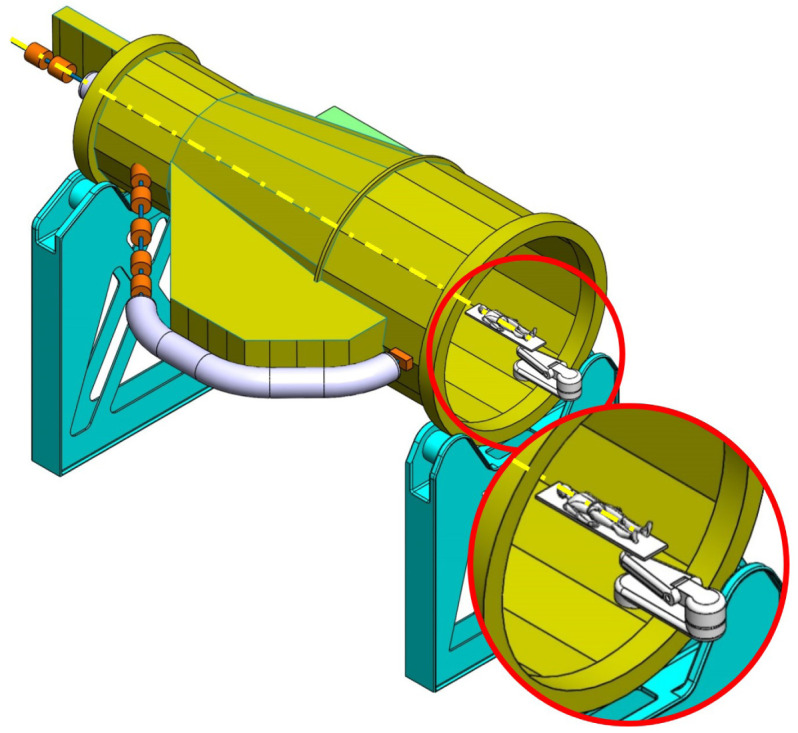	**Full Turn Gantry (FTG)** Two ground support balanced structure, driven by off-axis friction rollers, coupled to electric motor. Proposed to cover a full turn, minimizing bed couch movements. • ±180° deliverable treatment angle range. • External volume of construction: 28×28.5×27m ^3^ • Internal volume of construction: 16×20.5×20m ^3^ • Rotating mass about 120 ton. • Relevant moment of inertia: 700000 kgm ^2^

### Prospective scenarios

The scenarios that sufficiently satisfied requirements were more thoroughly investigated. They are the balanced variants shown in Table 2, their main properties being listed in Table 3.

**Table 3.** List of scenarios main properties.

	B-SIGRUM	SSG	SCSG	FTG	
**Treatment angle range**	±110	±110	±110	±180	deg
**External volume of construction**	21.5 × 28.5 × 27	22 × 28.5 × 30	21.5 × 28.5 × 27	28 × 28.5 × 27	m ^3^
**Internal volume of construction**	11.5 × 20.5 × 20	12 × 20.5 × 23	11.5 × 20.5 × 20	16 × 20.5 × 20	m ^3^
**Rotating mass**	70	70	120	120	ton
**Relevant moment of inertia**	500000	500000	700000	700000	kgm ^2^

The size of the support ring varies between SCSG and FTG, influenced by a different positioning of the patient couch which has to compensate the limited rotation of ±110° of the SCSG.

### Analysis of main parameters

Taking into account the technological requirements in Table 1 and the challenge related to costs, Table 4 lists a series of decisive parameters that have been proposed and analysed following the methodology explained in the homonym Section. The minimum clinical requirement of ±110° rotation is satisfied by all scenarios proposed, while only the FTG can cover the full ±180° rotation.

**Table 4.** List of key parameters.

Robustness of the design	
• Failure scenarios	Consider the possibility of the gantry to accelerate uncontrollably due to gravity or a failure of the motion system
• Possible safety brakes position	Evaluates the possibility to have safety brakes in strategical positions
• Number of systems to brake	Estimates the number of systems to brake
Size	
• Room space requirements	Evaluates the volume of the scenario
• Weight and complexity	
• Weight and inertia	Evaluates the weight and moment of inertia of scenarios
• Complexity	Evaluates the complexity of the assembly i.e. R&D needed for the supports
Performances: deformation and precision	
• Load properties	Express the max value of the load on the supports and if it is related to the angular position of the gantry during the treatment phase.
• Deformation performances (structure)	Evaluates the scenarios based upon displacements of the transfer line
• Improvement margin of deformation performances	Estimates the margin of mechanical optimization based upon knowledge
• Deformation performances (supports)	Assesses the deformation of the supports
• Tolerances	Estimates the difficulty in achieving tolerances in different scenarios
Costs	
• Cost of manufacturing components	Estimates relative costs of manufacturing components for each scenario
• Cost of the driving system	Estimates relative costs of the driving system
Environmental	
• Minimal energy consumption	Estimates the energy consuption

The main decisive parameters are explained in the following sections, those not described explicitly are thought to be deductible from the summary Table 6.

### Failure scenarios

Addressing failure scenarios, two key aspects have been considered: the acceleration due to gravity, and the malfunctioning of the driving system. The model of non-damped physical pendulum has been taken into account with Equation 1 in the case of an unbalanced gantry accelerating due to gravity:


$$\;\vartheta^{\prime \prime }=\frac{mgR_{COG}}{J}\sin\vartheta \;(1)$$

where: *m* is the mass of the gantry, *g* is the gravitational acceleration, *R _COG_* is the radial position of the center of gravity with respect to the rotation axis, *ϑ* is the angular position of the gantry (measured from the top vertical position) and *J* is the moment of inertia of the gantry. Equation 2 models the case of a malfunction of the driving system for balanced scenarios.


$$\;\vartheta^{\prime \prime }=\tau /J\;(2)$$

where *τ* is the torque applied to the gantry. The torque *τ* is assumed to be constantly applied and be twice the maximum nominal value of the torque.

**Figure 1.**
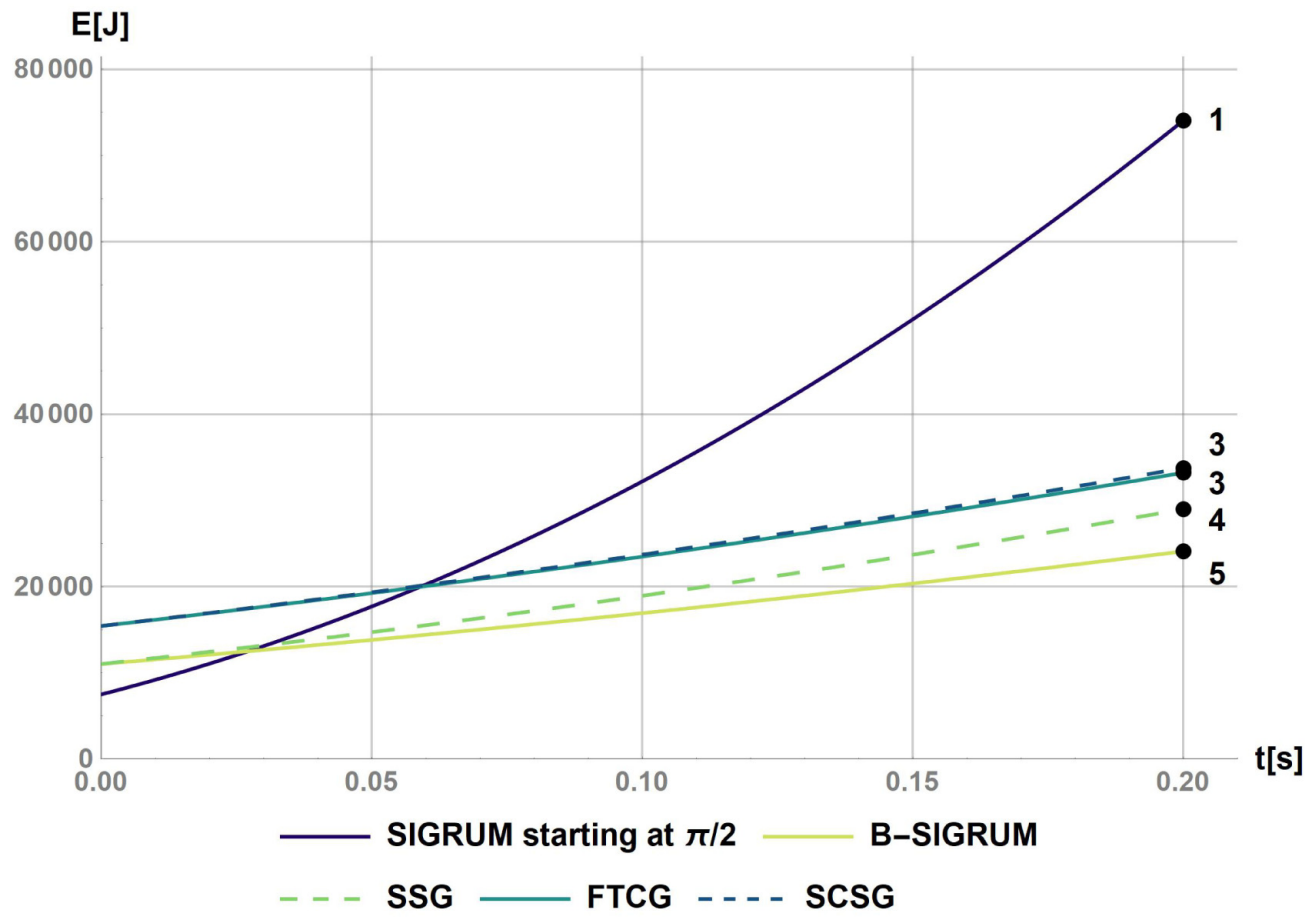
Kinetic energy of proposed scenarios within a reaction time of 0.2s. The values at t = 0.2s were used to rank the scenarios with the values reported on the figure.

The software Mathematica has been used to calculate the rotational kinetic energy for a base case (SIGRUM) and for the other balanced scenarios proposed (Python could have been used alternatively to obtain same results). This was done by solving numerically the differential Equation 1 and Equation 2 calculating the angular speed with a starting value of 0.21 rad s ^−1^ (as discussed above): results are reported in Figure 1. The value of 0.2 s represents the time before a collision of balanced structure with walls, assuming a clearance of 5° starting from the nearest available treatment position. This requirement shall also be used to design the treatment room environment avoiding dangerous collisions. Furthermore, the values of the kinetic energy after the reaction time have been used to rank structures in relation to the ‘failure scenarios’ parameter in Table 6.

### Deformation of the structure and its improvement margin

**Figure 2.**
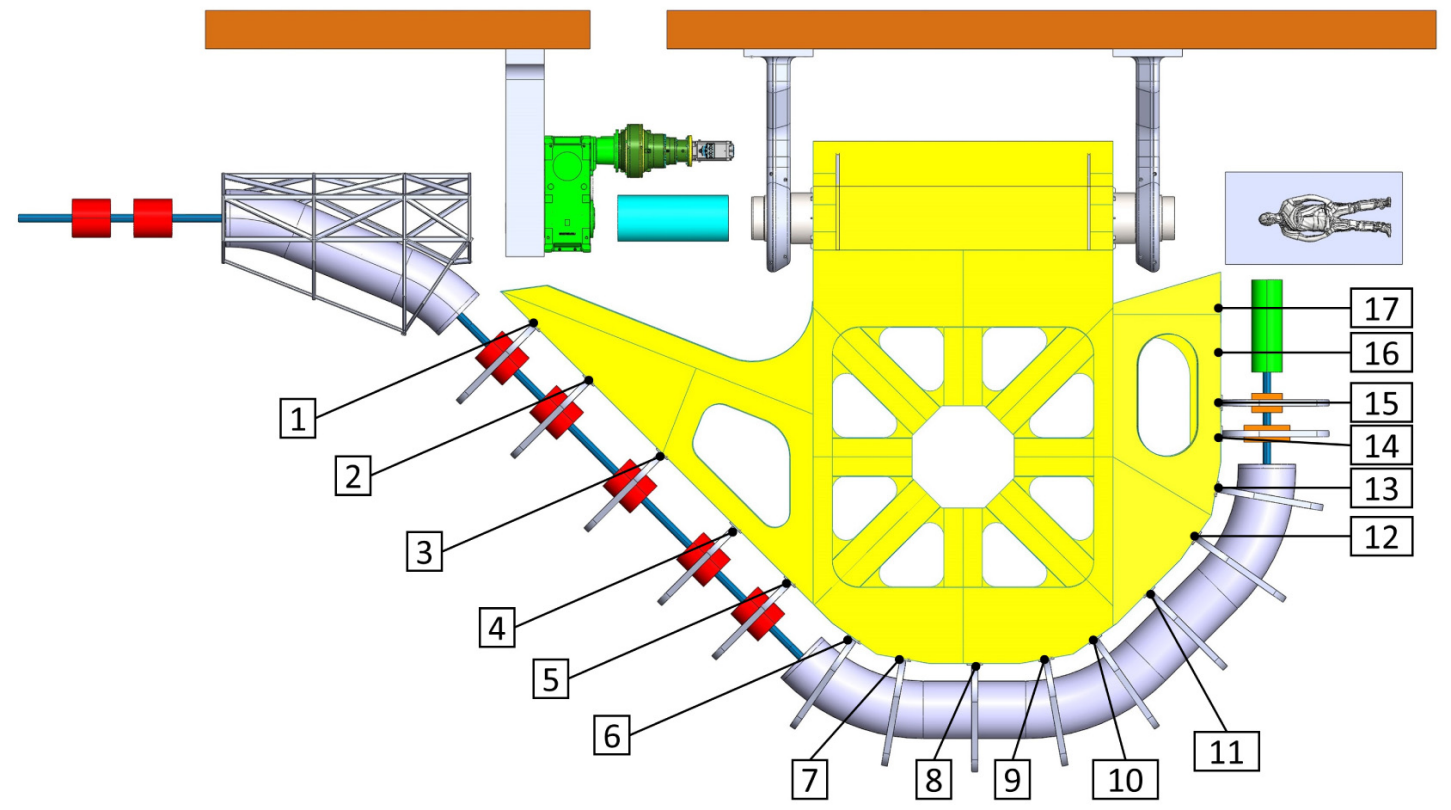
View of the mechanical optimized structure of the SIGRUM, numbers refer to analysed nodes for the displacement recovery.

**Figure 3.**
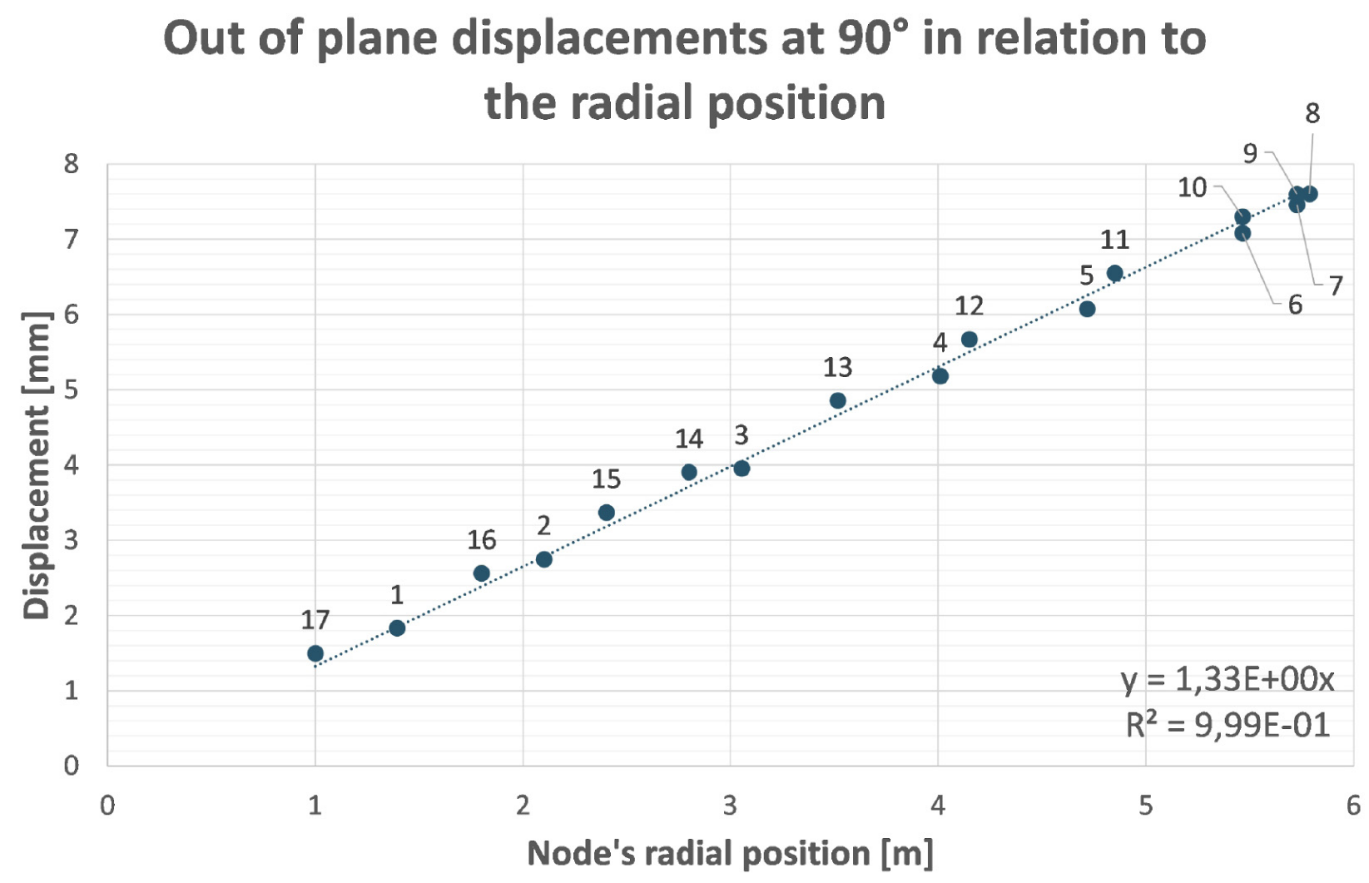
Out of plane (screen plane) displacement of analysed nodes ( Figure 2) in function of their radial position with respect to the rotation axis. The remaining displacement after recovery of its rigid component is the distance between nodes and the straight line through the origin.

Deformation performances of the structure were already addressed in the preliminary stage of the study: an estimation of displacement of transfer line elements was done for the SIGRUM in 15, 18. Whenever a requirement needs to be satisfied from the mechanical point of view it must be noted that stricter requirements (i.e. lower displacement) usually require higher stiffness, adding weight to the structure. Therefore, to achieve the compromise between stiffness and weight within the requirements set for this study, an innovative method of mechanical optimization to recover the rigid part of the displacement was proposed ^15, 18^, reducing the displacements by one order of magnitude (from several mm to less than a mm, see Figure 2 and Figure 3).

This optimization process has been proved to be particularly effective if applied to structures that behave like ‘cantilever’ beams (with a portion of structure hanging while one end is fixed). There is no reason to believe that this method is not applicable to B-SIGRUM with almost equal results. Furthermore, even if applicable this method has not yet been applied neither to SCSG nor to FTG, because the iterative mechanical optimization has not been performed. However, it is possible to assume that similar results can be reached for SCSG and FTG. Although the aforementioned method is not applicable to the SSG scenario, this is expected to benefit of similar displacement reduction thanks exclusively to its support configuration, acting like a simply supported beam (a structure supported at both ends, see Table 5).

**Table 5.** Comparison of maximum deflection between cantilever and simply supported beams. Where w is the weight per length unit, L is the length of the beam, E is the Young’s modulus of the material of the beam and I is the area moment of inertia of the cross section of the beam.

Supports configuration		Maximum displacement
Cantilever beam	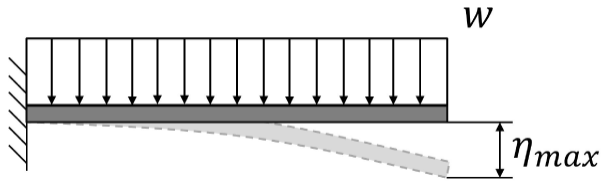	$\eta_{\max}=\frac{1}{8}\frac{wL^{4}}{EI}$
Simply supported beam	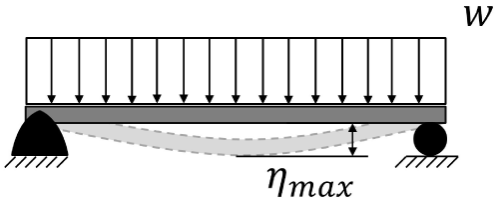	$\eta_{\max}=\frac{5}{384}\frac{wL^{4}}{EI}\approx 0.104\times \frac{1}{8}\frac{wL^{4}}{EI}$

**Table 6.** Summary table of scenario rankings with respect to each decisive parameter listed in Table 4.

**Robustness of the design**	Failure scenarios	Possible safety brakes position	Number of systems to brake	**Size**	Room space requirements	**Weight and complexity**	Complexity	Weight and inertia	**Performances: deformation and precision**	Tolerances	Deformation performances (structure)	Improvement margin of structure deformation	Deformation performaces (supports)	Load properties	**Costs**	Cost of manufacturing components	Cost of the driving system	**Environmental**	Minimal energy consumption	
	5	3	5		5		5	5		5	5	3	3	4		4	1		5	**SIGRUM**
	4	5	5		3		3	5		5	5	5	3	4		4	5		5	**SSG**
	3	4	5		5		4	3		4	5	4	4	3		2	4		4	**SCSG**
	3	4	5		1		4	3		4	5	4	5	5		2	4		4	**FTG**

All the scenarios have been ranked at the same level according to deformation performances ( Table 6). An estimated improvement margin of structure deformation has been defined. This evaluates the possible improvement in deformation analysis deriving from the mechanical optimization of a scenario with respect to the current knowledge. Therefore, SSG, SCSG and FTG are ranked better than B-SIGRUM, in relation to this parameter ( Table 6).

### Deformation of supports

Each scenario has a different combination of weight and support configuration, therefore side supports of B-SIGRUM are mainly subject to bending moments while bottom supports of FTG are loaded by compression forces. Since for balanced scenarios, during the treatment phase, these loads are not related to the angular position of the gantry, the supports will deform and the axis will be displaced by a constant value: this systematic error can be compensated finding a new origin for the patient couch or using adjustment systems (i.e. rails and shims between the supports and the gantry) to fine tune the position of the gantry in the assembly phase. As the loads are different for each scenario, side supports have been designed in SOLIDWORKS and studied with preliminary FEA in order to reach the same performances, namely the same horizontal and vertical displacement. Thus, differences in load values are decoupled from the evaluation of deformation performances and incorporated in the cost analysis (cost of components manufacturing): to achieve the same displacement under different loads, different amounts of material are needed. However, due to the different type of loads, adjustment systems have respectively different design complexity. Therefore, scenarios that have side supports have a lower rate than FTG which has two ground supports, as represented in Table 6.

### Geometrical tolerances

The errors due to tolerances can be compensated at different levels: between the gantry and its supports, between the gantry and cryostats, or fine tuning the position of magnets inside cryostats. Furthermore, the ability to reach strict tolerances is usually in competition with the size of the component to manufacture, therefore, it is believed that a relatively small shaft like for B-SIGRUM and SSG would be easier to manufacture within a given range of tolerance if compared with large rings of SCSG and FTG. It is underlined that tolerances were qualitatively discussed.

### Costs of components manufacturing

Costs of manufacturing components were estimated on the basis of previous knowledge on costs of main manufacturing processes that are necessary for this machine. The total cost of manufacturing components is composed by:


$$C_{\text{manufacturing}}=C_{\text{rot}-\text{structure}}+C_{\mathrm{supports}}+C_{\mathrm{counterweight}}\;(3)$$

where:

- *C*_rot-structure_ is the cost of the rotating structure manufactured in stainless steel.
- *C*_supports_ is the cost of support structures manufactured in construction steel.
- *C*_counterweight_ is the cost of the counterweight manufactured in lead.

The rotating structure and supports necessary for the functioning of each scenario were modeled and checked in simplified FEA to estimate the amount of material necessary to satisfy requirements. For example, for side supports, the amount of material was estimated to satisfy a maximum displacement of *L/*500- *L/*250 (common values for steel structures in engineering sound practice, where *L* is the span) and to have the same displacement between side supports of different scenarios (computed to be around 5mm or *L/*1800). The supports of magnets cryostats and instrumentation are related to the given transfer line layout (same for all scenarios), thus their cost can be neglected in the comparison. Therefore, Table 6 reports a ranking associated to relative costs between scenarios: it has been estimated that B-SIGRUM and SSG are lower in cost by a factor of two with respect to SCSG and FTG.

### Costs of the driving system

The costs of the driving system are related to the maximum power (25–40kW), torque and speed required by the load. These data were calculated by assuming the equation of motion of the gantry (the same for all scenarios) and as a function of the properties of the gantry (mass, inertia) and the geometric properties of the transmission (i.e for the FTG, the radius of the ring and rollers on which the gantry is supported). Scenarios are ranked mainly based on the output torque of the required gearbox. These costs are between one and two orders of magnitude smaller with respect to the cost of components manufacturing.

### Minimal energy consumption

**Figure 4.**
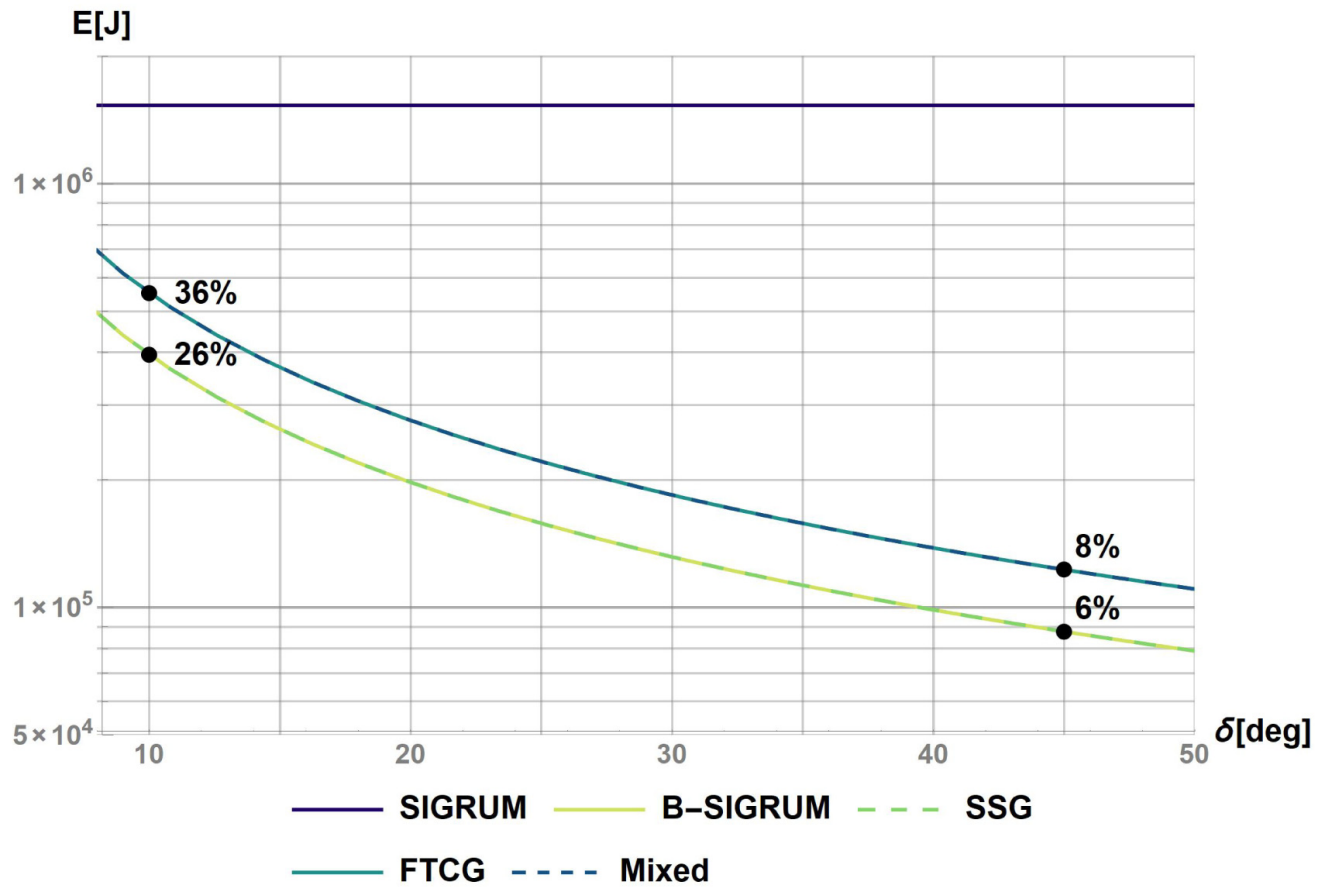
Work done by the motor (ideal) for a 180° rotation divided in equal δ angle steps.

The evaluation of the energy consumption was done analytically by calculating the work of the motor for a 180° rotation subdivided in equal *δ* steps. The results are reported in Figure 4: the percentages shown on the plot represent the energy ratios between different scenarios and the SIGRUM, the base example. The energy ratios are evaluated for two reference values, *δ* = 10° and 45°, respectively related to the minimum angle step between approved treatment angles, and a possible realistic number of treatment angles for a patient (4–5 angles). The expected energy ratio will range between these two values. In conclusion, the motion system of B-SIGRUM and SSG consume less energy with respect to SCSG and FTG, all four consuming less than the SIGRUM. Scenarios are rated accordingly in Table 6.

## Results of the comparative study

All proposed scenarios are safe, however, SSG is estimated to be the safest. B-SIGRUM and SSG are expected to be lower in cost by a factor of two with respect to SCSG and FTG. Statically balanced scenarios, by means of a relatively simple integrated counterweight, come with several advantages. Although some of the following considerations were not estimated quantitatively, they are not negligible:

1. Less kinetic energy during emergency braking, allowing to design less complex braking and driving systems,
2. Displacements of the rotational axis of the gantry not related to its angular position during the treatment phase: the related error can be compensated during the assembly phase once, reducing or excluding the need of a dedicated online control system,
3. Less cost per operation and environmental impact: the energy that the driving system need to supply is between 10% and 40% that for unbalanced ones,
4. Easier process for machinery certification according to the European applicable laws.

## Conclusions

Multiple scenarios were defined during discussions with experts of CERN EN-MME group, TE-MSC group, RTU and UNIBS in consultancy with CNAO experts in the framework of NIMMS and HITRI *plus* project. Main requirements ( Table 1) and challenges were acknowledged following HITRI *plus* ^16^ requirements and general designing rules for a machine of this kind. The comparative study has been performed in a comprehensive manner following a unified methodology providing objective results, summarized in Table 6. The main conclusions are:

1. Observations related to safety, deformation and precision performance, cost of operation, complexity of service systems, and potential certification costs, lead to the decision to focus only on statically balanced structures,
2. All four scenarios proposed are suitable for further development of gantry conceptual design,
3. All scenarios can be considered safe, however, SSG is estimated to be the safest,
4. B-SIGRUM and SSG are expected to be lower in costs with respect to SCSG and FTG by a factor of two.

## Data availability

Zenodo: Comparative Study on Scenarios for Rotating Gantry Mechanical Structures, https://doi.org/10.5281/zenodo.6516846 ^19^. Data are available under the terms of the Creative Commons Attribution 4.0 International license (CC-BY 4.0).
