# Supplementary material for: Comparative study on scenarios for rotating gantry mechanical structures
Source: Open Res Eur. 2023 Jun 27;2:55. Originally published 2022 May 6. [Version 3] doi: 10.12688/openreseurope.14683.3 (PMC11487233; doi:10.12688/openreseurope.14683.3)
Supplement: Peer review history

# Reviewer report: Reviewer response for version 3

**DOI:** 10.21956/openreseurope.17504.r33254

**Published:** 2023-06-30

**Authors:** Alexander Gerbershagen

**Affiliations:** 1 University Medical Center Groningen, Groningen, The Netherlands

The authors have made a minor rework of the article and have addressed some of the issues raised by the reviewer, which resulted in the overall improvement of the article. However, several specific issues have not been addressed. The reviewer recommends the authors either to address those, or to explain in the Amendments comment section why those cannot (or should not) be addressed. The comments are stated below:

 The size of the HIT gantry was added, but the size of HIMAC gantry was not, although they are listed in the same sentence. To help the authors - HIMAC gantry length is 16.9 m, the radius is 7.1 m (from Proceedings of EPAC2006, TUYFI01).

 In the environmental requirements, the decommissioning aspects are still not mentioned. The power consumption for gantry rotation is much less of a practical concern than 100s of tons of activated gantry material, which needs to be disposed after several decades of operation.

 All external volumes of construction summarized in Table 2 are above the envisaged dimensions of 20 m x 20 m x 20 m. Please address if that is an issue or not.

 “Aforementioned results have been obtained assuming to use stainless steel […]” – This is a costly solution, compared with the commercially available alternatives, which use iron. So it might be of advantage to make a simplified comparison of the mass you have stated (70/120 tons) multiplied with the present stainless steel price, and compare it with the price of iron-based existing gantries (e.g. HIT).

Is the study design appropriate and does the work have academic merit?

Yes

Is the work clearly and accurately presented and does it cite the current literature?

Yes

If applicable, is the statistical analysis and its interpretation appropriate?

Not applicable

Are all the source data underlying the results available to ensure full reproducibility?

Partly

Are the conclusions drawn adequately supported by the results?

Partly

Are sufficient details of methods and analysis provided to allow replication by others?

Yes

Reviewer Expertise:

Accelerator physics

I confirm that I have read this submission and believe that I have an appropriate level of expertise to confirm that it is of an acceptable scientific standard, however I have significant reservations, as outlined above.

# Reviewer report: Reviewer response for version 2

**DOI:** 10.21956/openreseurope.17058.r32106

**Published:** 2023-06-09

**Authors:** Alexander Gerbershagen

**Affiliations:** 1 University Medical Center Groningen, Groningen, The Netherlands

The article describes the analysis of the mechanical stability of several proposed gantry layouts for ion therapy. The article commences with a summary of efforts of CERN in developing ion therapy centres in CNAO and MedAustron. After a brief description of the overall concept, it focusses on the parameters the design aims to optimize. The authors describe the examined options and the methods used for analysis of designs’ mechanical suitability. However, no explicit effort in improving or optimizing the mechanical rigidity of the specific designs is described. Many aspects of the study remain qualitative, as designs are compared with each other, but not with the performance of the existing solutions or how close the expected performance relates to the clinical requirements. I suggest the authors to address the points mentioned below, as at the current stage the article, while addressing important issue with the correct methodology, lacks specific data to be of a high value for the readers.

 Specific comments:

 “Figures are expected to rise by more than 24% by 2035.” -> What of the figures mentioned above is expected to rise - the absolute numbers of cancer cases?

 “improvement of the Technology Readiness Level (TRL) of this design is the final outcome of the project, from the presently evaluated TRL 2” -> What TRL level is envisaged to be achieved at the end of the improvement process?

 Table 1 is not a list of requirements, but only a list of parameters targeted for the optimization, as no specific values are stated.

 Size of 20 m x 20 m x 20 m is stated, but it would be good for comparison to give the numbers for the existing systems (HIT, HIMAC).

 For the environmental requirements, the decommissioning aspects might need to be examined or at least mentioned. E.g. what materials would be used for the gantry construction and if those are recyclable, and how much activated material will be produced. Many old accelerators stand in the research centers for decades, because no plan of their decommissioning has been developed and their components cannot be recycled, as they have long-term activation.

 “Results have been summarized in a final Table”- no capital “T”, unless you are referring to a table with a specific number.

 The magnets referred to in [13] and [14] were first proposed by W. Wan ^1^. It might be appropriate to cite the article.

 All external volumes of construction summarized in Table 2 are above the envisaged dimensions of 20 m x 20 m x 20 m. Please address if that is an issue.

 “The scenarios that sufficiently satisfied requirements…” – what requirements did the other scenarios fail to satisfy? Only the lack of balance?

 “Aforementioned results have been obtained assuming to use stainless steel […]” – this sounds like an outstandingly costly solution, compared with the commercially available alternatives, which use iron. It obviously improves the rigidity of the system and decreases the overall weight, but the reason that it is not used for the commercial solutions is that the cost becomes unjustifiably high. So in the paper section describing the cost it would be good to see explicit numbers, at least based on the weight you have stated and the present stainless steel prices.

 Figure 3: Displacement of up to 8 mm is very notable. It would be good to have an estimate on what it implies for the beam position displacement. The displacement is maximal at the entrance region of the final dipole and might have a large impact on the beam deflection by this dipole. Also, will the dipole be flexible enough to accommodate these constant mechanical stresses without impact on its performance, short and long-term?

 Table 6: I suggest to put the name of the design into the first row.

Is the study design appropriate and does the work have academic merit?

Yes

Is the work clearly and accurately presented and does it cite the current literature?

Yes

If applicable, is the statistical analysis and its interpretation appropriate?

Not applicable

Are all the source data underlying the results available to ensure full reproducibility?

Partly

Are the conclusions drawn adequately supported by the results?

Partly

Are sufficient details of methods and analysis provided to allow replication by others?

Yes

Reviewer Expertise:

Accelerator physics

I confirm that I have read this submission and believe that I have an appropriate level of expertise to confirm that it is of an acceptable scientific standard, however I have significant reservations, as outlined above.

## References

[1] : Alternating-gradient canted cosine theta superconducting magnets for future compact proton gantries. *Physical Review Special Topics - Accelerators and Beams* .2015;18(10) : 10.1103/PhysRevSTAB.18.103501 10.1103/PhysRevSTAB.18.103501

# Response

**Published:** 2023-06-21

**Authors:** Luca Piacentini

**Affiliations:** Riga Technical University, Latvia

We would like to underline that the study at moment of publication was and still in its early stages, therefore it was (and still is) not possible to include studies detailed enough to fully reply to some comments. Regarding the comment on Environmental requirements, the transfer line, that is closer to the beam than any other structures would be the same for all gantry solutions. A study on the impact of radiation on the mechanical structure could be done in the future.   The article suggested has been mentioned in [13]. In general, it was not in the aim of this study to define in detail the technology to use. Indeed, as mentioned, the comparison of structures is relatively independent from the transfer line used.    The displacement for the structure is maximal at point 8, just after the middle of the transfer line  (Figure 2 and 3). The displacement of 8 mm alone is indeed notable. However, as cited in this article, studied in [15] and reported in Figure 3, the points where elements of the transfer line are connected lay around a common plane (dashed line). Around this common plane, the relative displacement between two adjacent elements of the line is of the order of tenths of mm. The impact on the beam is currently subject of research and was not in the aim of this article. Additionally, we would like to comment that between dipoles or cryostats are present flexible elements that allow for deformation. The length of life of these components could also be investigated in the future.

# Reviewer report: Reviewer response for version 1

**DOI:** 10.21956/openreseurope.15860.r29727

**Published:** 2022-08-01

**Authors:** Claire Z. Antoine

**Affiliations:** 1 CEA, Département des Accélérateurs, de la Cryogénie et du Magnétisme, Université Paris-Saclay, Gif-sur-Yvette, France

This paper describes a comparative study of a dozen different scenarios for the mechanical design of the gantries for an ion-therapy accelerator. Three main parameters were retained: minimization of the size and complexity of the set-up, without giving up on safety parameters, minimal energy consumption and treatment angle coverage with a minimum ± 110°. After a first screening, all unbalanced structures were eliminated because of their intrinsic danger, and only 4 balanced scenarios were retained and more thoroughly studied.

 This work is well written, in a language appropriate for the multiple backgrounds of the possible readers (accelerator scientists, mechanical engineers, therapeutic teams…). Some details from the exact calculation are not detailed and remain obscure to the non-specialist, but such details would be beyond the scope of this paper. References are proposed for those who want more details.

 I have only one critic, the distribution of table and figures, that are sometimes far from the corresponding paragraph, makes the reading (at least on screen) somewhat difficult.

 In particular, I propose that p6, where the first mention of table 6 appears, you replace “deductible from the summary Table 6” by “deductible from the summary Table 6 that will appear at the end of the comparative study (or something similar), and that Table 6 indeed appears at the end of the study (e.g. just before the conclusion). If some effort can be made to put other figures and tables closer from the text where they are mentioned, it will improve the readability, but it still understandable like it is.

Is the study design appropriate and does the work have academic merit?

Yes

Is the work clearly and accurately presented and does it cite the current literature?

Yes

If applicable, is the statistical analysis and its interpretation appropriate?

Not applicable

Are all the source data underlying the results available to ensure full reproducibility?

Yes

Are the conclusions drawn adequately supported by the results?

Yes

Are sufficient details of methods and analysis provided to allow replication by others?

Partly

Reviewer Expertise:

I have been working in the field of superconducting accelerators for more than 30 years. Although I am not an mechanical engineer, I was already familiar with the the issues for gantry design.

I confirm that I have read this submission and believe that I have an appropriate level of expertise to confirm that it is of an acceptable scientific standard.
